# Egg white hydrolysate improves fatigue due to short‐term swimming load test in mice

**DOI:** 10.1002/fsn3.810

**Published:** 2018-10-10

**Authors:** Ryosuke Matsuoka, Mamoru Kimura, Shinya Uno, Hiroyuki Shidara, Masaaki Kunou

**Affiliations:** ^1^ R&D Division Kewpie Corporation Tokyo Japan

**Keywords:** antifatigue, antioxidant, egg white hydrolysate, swimming endurance

## Abstract

We studied the effect of egg white hydrolysate (EWH) on swimming endurance in mice. 7‐week‐old male ddY mice (28–30 g) were divided into three groups and fed an AIN‐93G diet supplemented with casein (*n* = 8), EWH (*n* = 7), or egg white protein (EWP,* n* = 8) for 14 days. From day 11, the mice underwent a swimming test daily with a weight load equivalent to 10% of their body weight, and the lengths of time they swam were recorded. Blood was sampled for testing on the last day of the study. We observed that increases in the swimming duration through day 14 were significantly greater in the EWH group than in the casein group (*p* = 0.049). As a factor underlying this, the hexanoyl‐lysine level in blood was confirmed to be decreased in the former group (*p* = 0.013). These findings indicate that consumption of EWH extended the swimming duration and suggest the mechanistic involvement of an antifatigue effect mediated by its antioxidant activity.

## 1 INTRODUCTION

A significant positive correlation between protein intake and muscle mass has been reported (Houston et al., 2008). Sarcopenia is a condition caused by the decline in muscle and bone mass. In particular, age‐related loss of muscles cannot be reversed by any existing drugs and must be treated with nutrition and physical exercise. Protein is a nutrient that builds muscles, and its intake is known to be significantly associated with muscle mass. However, it is quite challenging for the elderly to consume a large amount of protein, and they need sources of high‐quality protein. Meanwhile, strenuous exercise produces excessive free radical in muscle tissues (Murakic‐Sposta et al., 2015), while causing muscles to be damaged or fatigued (Nosaka & Newton, 2002; Nosaka, Newton, & Sacco, 2002). This can lead to reduced physical activities, which may turn into a vicious cycle of muscle mass decline. Therefore, early recovery from muscle fatigue is critical in maintaining and enhancing physical health.

The early recovery from muscle damage is significant not only for the elderly, but also for athletes for continuing their training. There are a number of means to counter such damage, which include massage, stretching, and improved nutrition through a balanced diet. And, for a balanced diet to work efficiently, it must include high‐quality sources of protein.

Previous studies involving human subjects have found that EWH reduce muscle damage in long‐distance runners (Sugiyama et al., 2016).

The egg white's protein component has been reported to have a high amino acid score and a high net protein utilization rate, and thus, it represents a good source of protein (Matsuoka, Takahashi, Kimura, Masuda, & Kunou, 2017; Sheffner, Eckfeldt, & Spector, 1956). Egg white proteins (EWP) are reported to decrease cholesterol (Matsuoka, Usuda, Masuda, Kunou, & Utsunomiya, 2017; Matsuoka et al., 2008; Matsuoka et al., 2014), improve visceral fat obesity (Matsuoka et al., 2017; Matsuoka, Shirouchi et al., 2017) facilitate iron absorption (Kobayashi, Kido, & Nakabou, 2007) and, when combined with exercise, increase muscle (Kato, Sawada, Numao, & Suzuki, 2011). Egg white hydrolyzed to peptides by digestive enzymes is expected to be absorbed more quickly and have novel physiological functions. Previously, egg white‐derived peptides were reported to decrease blood pressure (Miguel, López‐Fandiño, Ramos, & Aleixandre, 2005).

Egg white is characterized by high levels of sulfur‐containing amino acids and branched‐chain amino acids (BCAAs) (WHO, 1985). Because glutathione is produced in vivo from sulfur‐containing amino acids (Seligson & Rotruck, 1983), egg white can be expected to have antioxidant effects that are reportedly linked to antifatigue effects and the prevention of various diseases such as arteriosclerosis (Steinberg, Parthasarathy, Carew, Khoo, & Witztum, 1989). Moreover, BCAAs have been reported to ameliorate muscle fatigue (Matsumoto et al., 2009). Their concentration in the blood increases after ingesting an EWP preparation and decreases after exercise (Kato, Numao, Miyauchi, & Suzuki, 2010). Therefore, an antifatigue effect mediated by BCAAs can also be expected.

Although there are no reports on the antifatigue effects of proteins, such reports have been published for egg white‐derived peptides. Imidazole peptides from chicken meat have been reported to exhibit antioxidant effects, thus imparting antifatigue effects (Harada et al., 2002). Whey‐derived peptides have been reported to extend swimming duration in mice via radical‐scavenging and iron‐chelating actions (Liu, Wang, & Zhao, 2004).

Davalos, Miguel, Bartolome, & Lopez‐Fandino (2004) have reported the antioxidant effects of egg white hydrolysate (EWH). Antioxidant effects of pepsin‐hydrolyzed egg white have been reported in vivo; thus, it is associated with antifatigue effects (Sun, Niu, Yang, lin, Luo, & Ma, 2014). Particularly, in athletes, accumulated physical fatigue affects their performance the following day, and it can lead to injuries in worst‐case scenarios. Food product development with antifatigue effects is thus likely to be useful for athletes.

Although antifatigue effects have been reported for EWH, reports on this issue are limited to studies using peptides prepared by enzymatic degradation. Proteins digested with enzymes and peptides have beneficial effects on health; however, they taste bitter and unpleasant as consumable food. We thus developed a new EWH preparation with decreased bitterness by collecting water‐soluble fractions from enzyme‐digested egg white. In this study, we assessed the influence of the EWH preparation on swimming endurance in mice.

## 2 MATERIALS AND METHODS

### 2.1 Materials

Egg white hydrolysate, primarily developed as pathological nutrition food, was used and was prepared by successively heating egg white under alkaline conditions, followed by enzyme treatment with a neutral protease, heating to inactivate the protease, filtration to remove insoluble matter, and then spray‐drying (Watabe, 2013). The resulting EWH had a mean molecular weight of 700 Da and contained the following nutrients: 71.9 g/kg of protein, 0.9 g/kg of fat, 11.9 g/kg of minerals, and 10.8 g/kg of carbohydrate. Table 1 shows the amino acid composition. The EWP preparation used was dried egg white K type (CS No. 2) from Kewpie Corporation. Casein was purchased from Oriental Bioservice (Kyoto, Japan).

**Table 1:** Amino acid composition of egg white hydrolysate (mg/g N)

Amino acid	Egg white hydrolysate
Cys	117
Met	212
Thr	312
Val	442
Ile	302
Phe	291
Leu	521
Tyr	253
Lys	438
His	139
Try	85
Asp	734
Ser	449
Glu	971
Gly	255
Ala	397
Arg	387
Pro	228

### 2.2 Animals and diets

In this study, 6‐week‐old male ddY mice (Japan SLC, Shizuoka, Japan; 28–30 g, *n* = 30) were used. The ddY mice were used because this strain has been frequently used for animal tests in sports science and antifatigue research and had a track record of successful use in the present test system (Kamakura, Mitani, Fukuda, & Fukushima, 2001; Nakagawasai et al., 2013; Osada, Komai, Sugiyama, Urayama, & Furukawa, 2004). The mice were housed in stainless steel cages placed in an environment with lights on between 7:00 and 19:00, a temperature range of 20–26°C, and a humidity of 40%–70%. Their diet was prepared in accordance with the AIN 93G composition (Reeves, Nielsen, & Fahey, 1993). The reason for using the AIN‐93G diet was the latest base diet. The diet was supplemented with 20% casein, EWP, or EWH. Other components were cysteine (0.3%), α‐cornstarch (13.2%), cellulose (5%), sucrose (10%), mineral mix (AIN‐93G; 3.5%), vitamin mix (AIN‐93; 1%), soybean oil (7%), choline bitartrate (0.25%), tert‐butylhydroquinone (0.0014%), and β‐cornstarch (the remainder).

After 7 days of habituation, 30 mice were divided into three equal groups each to be fed a casein‐containing diet, EWH‐containing diet, or EWP‐containing diet. The mice were allowed to freely ingest the assigned diet and distilled water for 14 days. They underwent swimming time assessments from day 11 onward, and the swimming time periods were recorded.

We chose to mix a dose in feed because this test was a food evaluation and we wanted to conduct the test in a condition as natural as possible. In some feeding tests, food intake does not stabilize until animals get used to depending on the type of diet. Since exercise loading before food intake stabilizes increases variability of the food intake, no exercise load was applied before the food intake stabilized in the present test. In this test, the food intake levels of the animals became stable on day 8, and swimming time evaluation was started 3 days after the food intake stabilized (day 11) and conducted for 3 days thereafter.

After completing the swimming test on the last day of the study, the mice were sacrificed by drawing blood from the vena cava under anesthesia using pentobarbital (Nembutal; Sumitomo Dainippon Pharma, Tokyo, Japan). Blood samples were centrifuged at 1,700 *g* for 15 min and then sera were collected. Serum samples were stored at −80°C until analyses. This study was approved by the Animal Care and Use Committee of the Center of Japan Biological Chemistry & Co. and was conducted at the Center of Japan Biological Chemistry & Co. This experiment was performed under the guidelines for Animal Experiments, Law No. 105 and Notification No. 6 of the Government of Japan.

### 2.3 Swimming time assessment

An acrylic resin cylinder (19‐cm diameter, 50‐cm height) containing tap water (20‐cm deep) at 23–24°C was used. With a weight corresponding to 10% of its body weight, each mouse was made to swim in the cylinder, and swimming time was defined as the length of time until the mouth and nose of the mouse remained continuously underwater for 10 s (Zhang et al., 2011).

### 2.4 Blood analysis

In vena cava serum samples, lactic acid was measured with Lactic Assay Kit II (Bio Vision, Inc., Milpitas, CA, USA), creatinine kinase with CPK II Test Wako (Wako Pure Chemical Industries, Tokyo, Japan), and hexanoyl‐lysine using a Hexanoyl‐lysine Measurement Kit (Nikken SEIL, Tokyo, Japan) (Allain, Henson, Nadel, & Knoblesdorff, 1973; Marbach & Weil, 1967; Sakai et al., 2014).

### 2.5 Statistical analysis

Test results are presented as mean ± standard error. Statistical analyses were performed by one‐way analysis of variance followed by Tukey–Kramar test if a significant difference was detected in the former analysis. A difference was considered significant when the hazard rate was <5%. Statistical analyses were performed using the computer software Dr. SPSS II for Windows (SPSS, Tokyo, Japan).

## 3 RESULTS

### 3.1 Growth parameters

Here, ad libitum feeding was used, resulting in mean food intake being significantly less in the EWH group (5.3 ± 0.1 g) than in the casein group (5.8 ± 0.2 g) or the EWP group (5.7 ± 0.2 g). To minimize any effect of different protein intake on the swimming time, we excluded two mice each with the highest intake in the casein and EWP groups, and two mice with the lowest intake in the EWH group. In the EWH group, there were three mice with the second lowest food intake. Of these, the one with the lowest weight was excluded, because the mean body weight in the EWH group (41.4 g) was less than that in the other two groups (casein group, 43.3 g; EWP group, 42.3 g). In addition, one mouse in the EWH group had a mean swimming time of 73 s over 4 days, which was considerably shorter than the mean swimming time (195 s) of all other mice. This mouse was considered incapable of swimming and was thus excluded from the analyses. The analysis set, therefore, included eight animals from the casein group, seven animals from the EWH group, and eight animals from the EWP group. No significant differences in body weight gain, dietary intake, and feed efficiency were found among the three groups (Table 2).

**Table 2:** Growth parameters in mice

	Initial body weight (g)	Final body weight (g)	Body weight gain (g/day)	Dietary intake (g/day)	Food efficiency
Casein	35.6 ± 0.6	42.3 ± 0.6	0.48 ± 0.04	5.58 ± 0.17	0.09 ± 0.01
EWH	34.3 ± 0.4	41.6 ± 0.8	0.52 ± 0.06	5.30 ± 0.10	0.10 ± 0.01
EWP	34.6 ± 0.7	41.7 ± 0.8	0.51 ± 0.05	5.55 ± 0.16	0.09 ± 0.01

*Notes*

Mean ± *SE* of 7–8 mice.

EWH: egg white hydrolysate; EWP: egg white protein.

### 3.2 Swimming time assessment

**Figure 1:**
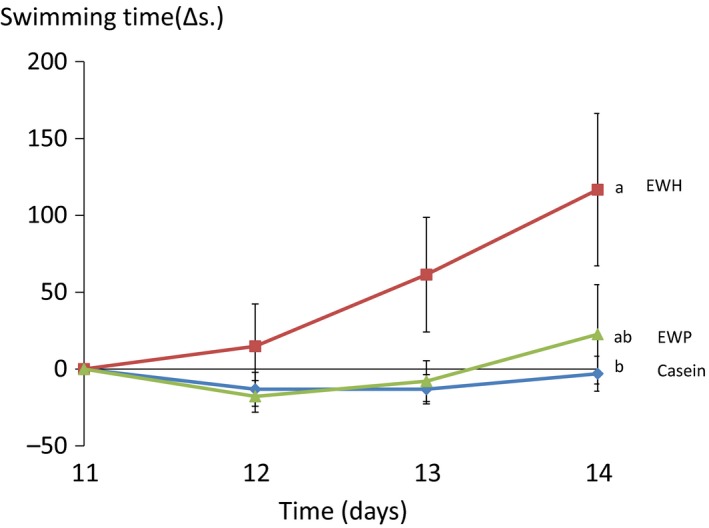
Swimming time in ddY mice fed casein, egg white hydrolysate, or egg white protein. EWH: egg white hydrolysate, EWP: egg white protein. Mean ± *SE* of 7–8 mice. Different letters represent significant differences (*p* < 0.05)

The results on swimming time for mice that received casein, EWH, and EWP are shown (Figure 1). At the commencement of the study, the mean swimming time was 132 ± 16 s in the casein group, 151 ± 21 s in the EWH group, and 151 ± 21 s in the EWP group, showing no significant differences among the three groups. Although the data on day 11 did not show significant intergroup differences, the swimming time in the casein group tended to be shorter than that in the EWH group or the EWP group. Therefore, we expressed the swimming time data as Δ with the swimming time on day 11 being defined as 0. The Δ swimming time on day 14 in the EWH group was found to be significantly greater than that in the casein group or the EWP group (*p* = 0.049).

### 3.3 Blood tests

Blood analyses were performed on mice immediately after swimming. Blood lactic acid levels in the casein, EWH, and EWP groups were 1141 ± 9, 1256 ± 16, and 1225 ± 16 nmol/ml, respectively, indicating that levels in the EWH and EWP groups were significantly higher than the level in the casein group. Blood creatinine phosphokinase concentrations in the casein, EWH, and EWP groups were 20.6 ± 1.9, 22.5 ± 1.3, and 22.8 ± 0.8 IU/l, respectively, indicating no significant differences among the three groups. Blood hexanoyl‐lysine levels in the casein, EWH, and EWP groups were 89.8 ± 26.2, 20.1 ± 4.3, and 26.6 ± 8.3 nmol/l, respectively, indicating that the EWP and EWH groups of mice had significantly lower levels than the casein group.

As these parameters are likely to be affected by swimming duration, concentrations per unit swimming time were calculated (Table 3). The results showed no intergroup differences in per swimming time values of serum lactic acid and creatinine kinase levels. The hexanoyl‐lysine concentration per swimming time in the EWH and EWP groups was significantly higher than that in the casein group (*p* = 0.013 and *p* = 0.022, respectively).

**Table 3:** Serum antifatigue parameters in mice

	Lactic acid (mg/ml/min swimming time)	CPK (mg/ml/min swimming time)	HSL (mg/ml/min swimming time)
Casein	478 ± 32	8.68 ± 1.10	35.2 ± 10.0^a^
EWH	422 ± 87	7.62 ± 1.79	6.39 ± 1.48^b^
EWP	450 ± 31	8.27 ± 0.49	9.51 ± 0.27^b^

*Notes*

Mean ± *SE* of 7–8 mice. Different letters represent significant differences (*p* < 0.05).

EWH: egg white hydrolysate; EWP: egg white protein.

## 4 DISCUSSION

The results of this study confirmed that EWH exhibited an antifatigue effect via its antioxidant activity. In general, intensive exercise results in the generation of reactive oxygen species, which have been reported to damage DNA and cells and cause fatigue (Kawanishi, Hiraku, & Oikawa, 2001; Kobayashi, Oikawa, Umemura, Hirosawa, & Kawanishi, 2008).

The results also confirmed an in vivo antioxidant effect of EWH, as indicated by the decreased blood hexanoyl‐lysine levels after receiving EWH. Furthermore, the study confirmed an in vivo antioxidant effect after swimming in the EWP group, but only the EWH group showed an extended swimming time.

It has been reported that reactive oxygen species are generated in the body after performing intensive exercise. This study showed a significantly longer swimming time in the EWH group than in the casein group or the EWP group. Therefore, we calculated hexanoyl‐lysine levels per unit swimming time and found a significantly lower value in the EWH group than in the casein group, with no significant difference between the EWP and casein groups. This finding confirmed a more potent in vivo antioxidant effect of EWH.

Some reports have documented the antifatigue effect of peptides, but no such reports are available for EWP itself. This is attributable to differences in the amino acid absorption rate. It is clear that low‐molecular‐weight peptides have higher absorption rates than high‐molecular‐weight proteins (Hara, Funabiki, Iwata, & Yamazaki, 1984; Matthews & Adibi, 1976).

The observed effect was assumed to be attributable to a decreased physical burden because EWH ingested immediately after swimming quickly migrated into target organs such as muscles and could quickly decrease reactive oxygen species therein. Another contributing factor may be the rapid supply of amino acids required to repair muscle cells disrupted during exercise. These findings may explain the observed significant differences in serum creatinine kinase levels among the three groups. The antifatigue effect has also been reported for pepsin digests from EWP, and the absorption rate was considered a likely contributing factor (Sun et al., 2014). Therefore, this difference in absorption rates may be involved in the various increases in swimming time. And we think this is a subject for further studies. Egg white is a quality source of protein, and muscle enlargement has been reported to occur when it is ingested in addition to exercise (Kato et al., 2011). A muscle‐enlarging effect may also be expected for EWH, although we could not address this issue in the present study.

The EWH used in this study was prepared by degradation with a neutral protease, which is not a physiological digestive enzyme. Therefore, the EWH likely comprised peptides with sequences different from those of products of EWP digestion in the body, and it is also possible that such peptides are involved in the antifatigue effect. Further elucidatory studies are required to shed light on this issue.

Regarding active ingredients in this study, we think that they may include antioxidant peptides, since we confirmed that the egg white hydrolysate itself had the antioxidant effect, although the process of digestion and absorption was not taken into consideration. In addition, egg white peptides contain many sulfur‐containing amino acids and BCAA (Table 1). Sulfur‐containing amino acids can be expected to exert the antioxidant effect, since they are source materials for producing glutathione (Seligson & Rotruck, 1983). BCAA has been reported to improve muscle fatigue (Matsumoto et al., 2009), and although it is possible that they caused muscle hypertrophy, it is unlikely that muscle hypertrophy occurred in a short‐term test like the present test.

Lysine has been reported to have an antifatigue effect and is also a component of hexanoyl‐lysine which is used in this study as an indicator of antioxidative effects in vivo. We thus calculated the amount of lysine intake based on the amino acid composition value of EWH in this study, as well as those of casein and EWP reported elsewhere (Matsuoka, Shirouchi et al., 2017) The results were as follows: 1.37 g/100 g in the casein diet, 1.01 g/100 g in the EWH diet, and 1.23 g/100 g in the EWP diet. In other words, the amount of lysine intake was lowest in the EWH diet, followed by the EWP diet and the casein diet. This has suggested that lysine intake was more closely related to the antioxidative effects in vivo rather than to the extended swimming time. Therefore, we studied the correlation between lysine intake and the serum concentration of hexanoyl‐lysine, which turned out to be nonsignificant (*r* = 0.256, *p* = 0.222). As a follow‐up, we checked the correlation between the change in swimming time and lysine intake, and also found it to be nonsignificant (*r* = 0.303, *p* = 0.160). These indicate that the results of our study were not affected by lysine intake.

In a previous study, antioxidant and antifatigue effects were observed with pepsin‐degraded products of EWP at a low dose of 0.2 mg/g/day (Sun et al., 2014). As pepsin is a typical enzyme found in the stomach, it is reasonable to expect effects similar with those of the ingestion of unprocessed EWP, although it may be less effective. However, virtually no antioxidant effect or extension of swimming time was observed in this study for mice that received approximately 1 g/day (approximately 25 mg/g/day) of the EWP preparation. There are two potential reasons for this.

First, the pepsin digest of egg white used previously was prepared by subjecting heat‐treated egg white to pepsin (Sun et al., 2014). In contrast, the EWP preparation used in the present study did not undergo any heat treatment. Ovalbumin, a protein in egg white, is known to be susceptible to hydrolysis by pepsin only when it is heated in advance (Sakai, Ushiyama, & Manabe, 1999). Therefore, products from pepsin treatment of heat‐treated ovalbumin may contain potent antioxidant peptides. The EWH used in our study was also prepared from egg white heated prior to the enzyme treatment.

Second, as opposed to the ad libitum feeding used in the present study, oral administration was used in the previous study, which likely allowed effective scheduled administration of the pepsin‐treated preparation of egg white (Sun et al., 2014). EWP do not exhibit antifatigue effects unless an appropriate dosing schedule is used. The EWH used in this study, on the contrary, resulted in antioxidant effects and extension of swimming time under ad libitum feeding conditions, suggesting that it has beneficial effects regardless of the feeding schedule.

In this study, blood lactic acid levels in the EWP and EWH groups were significantly higher than those in the casein group. It is generally considered that lactic acid is generated as a result of fatigue (Brooks & Gladden, 2003). However, this notion has been questioned by some studies, and the details remain to be elucidated further (Gladden, 2004). This study also showed that EWH extended the swimming time despite a significant increase in lactic acid in blood compared with the level observed for animals receiving casein, suggesting that an elevated lactic acid level did not influence the antifatigue effect in this study. The absence of significant intergroup differences in blood lactic acid per swimming time suggests that the increased lactic acid levels after swimming observed in the EWP and EWH groups reflect effects from an increased swimming time rather than dietary effects.

It has been demonstrated in mouse experiments that the ingestion of 500 mg/kg/day (0.5 mg/g/day) of an imidazole dipeptide produced antifatigue effects (Harada et al., 2002). The EWH in this study showed an antifatigue effect at an approximate daily intake level of 25 mg/g. The daily intake of 200 mg of imidazole dipeptide in humans has been reported to be effective for achieving an antifatigue effect, albeit weakly, in those experiencing tiredness in daily life (Hayami, Fukuda, & Yamamoto, 2009). Although the minimum effective dose for the antifatigue effect should be determined empirically in human studies, a simple calculation using these values allowed us to estimate it as approximately 10 g per day of EWH.

Issues associated with the consumption of egg white include allergic reactions. Given the fact that EWH is prepared by the enzymatic degradation of egg white, it seems reasonable to expect that EWH would be hypoallergenic. Patients with egg allergies may thus be able to use EWH safely, although close monitoring may still be required.

Problems associated with peptides include bitterness and insoluble matter that can affect suitability for food processing. The peptide preparation used in this study comprises a combined water‐soluble fraction from enzyme‐digested egg white. Because peptides constitute the main component, EWH is resistant to heat, and it is possible for an antifatigue dose of 10 g per day to be taken by supplementing beverages with EWH. Furthermore, the EWH used in this study has a decreased level of bitterness and can be ingested without adding any masking agent. Thus, EWH appears to have a wide variety of food applications.

## 5 CONCLUSION

Although some issues remain to be addressed in future studies, the results presented here demonstrate that EWH extended the swimming time in mice and suggest the mechanistic involvement of antifatigue effects via antioxidant activity.

## CONFLICT OF INTEREST

The authors have no conflict of interests to declare.

## ETHICAL APPROVAL

This study was approved by the Animal Care and Use Committee of the Center of Japan Biological Chemistry & Co. and was conducted at the Center of Japan Biological Chemistry & Co. (authorization no. JBS‐10‐MOPP‐633; September 30, 2010). This experiment was performed under the guidelines for Animal Experiments, Law No. 105 and Notification No. 6 of the Government of Japan.

## AVAILABILITY OF DATA AND MATERIALS

The dataset supporting the conclusions of this article is included within the article.
